# TNF and TNF Receptor Superfamily Members in HIV infection: New Cellular Targets for Therapy?

**DOI:** 10.1155/2013/484378

**Published:** 2013-12-19

**Authors:** Amit Kumar, Wasim Abbas, Georges Herbein

**Affiliations:** ^1^Department of Virology, University of Franche-Comte, CHRU Besançon, UPRES EA4266 Pathogens & Inflammation, SFR FED 4234, 25030 Besançon, France; ^2^Department of Virology, University of Franche-Comte, Hôpital Saint-Jacques, 2 Place Saint-Jacques, 25030 Besançon Cedex, France

## Abstract

Tumor necrosis factor (TNF) and TNF receptors (TNFR) superfamily members are engaged in diverse cellular phenomena such as cellular proliferation, morphogenesis, apoptosis, inflammation, and immune regulation. Their role in regulating viral infections has been well documented. Viruses have evolved with numerous strategies to interfere with TNF-mediated signaling indicating the importance of TNF and TNFR superfamily in viral pathogenesis. Recent research reports suggest that TNF and TNFRs play an important role in the pathogenesis of HIV. TNFR signaling modulates HIV replication and HIV proteins interfere with TNF/TNFR pathways. Since immune activation and inflammation are the hallmark of HIV infection, the use of TNF inhibitors can have significant impact on HIV disease progression. In this review, we will describe how HIV infection is modulated by signaling mediated through members of TNF and TNFR superfamily and in turn how these latter could be targeted by HIV proteins. Finally, we will discuss the emerging therapeutics options based on modulation of TNF activity that could ultimately lead to the cure of HIV-infected patients.

## 1. Introduction

The term tumor necrosis factor (TNF) came into existence in 1975 with the work of Carswell and colleagues while studying hemorrhagic necrosis by endotoxin [1]. It was described as a host factor, a glycoprotein induced in response to endotoxin that has the capacity to kill the tumor. As the time progressed, TNF was realized to be rather a member of a superfamily that governs by binding to their receptors. TNF and TNF receptors (TNFR) are growing members of ligand and receptor superfamily that regulate several complex signaling pathways leading to apoptosis, inflammation, cellular differentiation, and antiviral state. The first member of TNF superfamily discovered is TNF-alpha (old name cachectin), a pleiotropic proinflammatory cytokine that plays pivotal role in several pathological conditions due to inflammation and infection [2]. Role of TNF in malignancies and inflammation conditions like arthritis have been reviewed extensively elsewhere [3–5].

Till date TNF superfamily comprises of 19 ligands and 29 receptors [4]. All members are proinflammatory in nature playing diverse roles [4]. Most of the members act like dual edge sword, both beneficial and in adverse role [4, 6, 7]. First two members of TNF ligand (TNFL) superfamily were TNF-alpha and TNF-beta, recognized first at protein level followed by identification of their respective cDNAs, while rest of the members were discovered based on cDNA sequence homology [4, 8, 9]. All members of TNF superfamily and their receptors have been comprehensively reviewed recently [4]. Besides TNF-alpha and TNF-beta, TNFL superfamily include CD40L, CD30L, FasL, TNF-related apoptosis-inducing ligand (TRAIL), lymphotoxin-beta (LT-beta), LIGHT, receptor activator of NF-kappaB ligand (RANKL), 4-1BBL, CD27L, OX40L, TNF-related weak inducer of apoptosis (TWEAK), a proliferation-inducing ligand (APRIL), B-cell activating factor (BAFF), vascular endothelial cell-growth inhibitor (VEGI), ectodysplasin A (EDA)-A1, EDA-A2, and GITRL [4, 10]. TNFR superfamily includes TNFR1, TNFR2, LT-betaR, OX40, CD27, CD40, CD30, 41-BB (CD137), Fas, TRAILR1 (DR4), TRAILR2 (DR5), TRAILR3, TRAILR4, OPG, RANK, Decoy (DC) R3, TWEAKR, NGFR, Transmembrane Activator and CAML interactor (TACI), BAFFR, LIGHTR (HVEM), DR3, glucocorticoid induced TNF receptor (GITR), EDAR, XEDAR, TROY, RELT, DR6, and B-cell maturation protein (BCMA) [4, 7]. Extracellular domains of TNFR family members have a typical cysteine rich motif. However, intracellular domains show variation contributing to diverse functions [7, 11]. On the basis of presence or absence of 45 amino acid long regions in their intracellular domain called death domain, TNFR members are categorized into two groups [4]. Presence of death domain is critical for the interaction with other proteins leading to cell death. For example, TNFR1 possess this death domain on the other hand, TNFR2 does not have the death domain.

Number of TNF ligand versus receptor suggests that some of the ligands interact with more than one receptor to achieve their goal [4]. TNF ligands and receptors are mostly expressed by immune cells. However, under certain pathophysiological conditions their presence has been documented in other cell types as well.

## 2. TNF-Alpha-Mediated Cell Signaling: An Overview

Most extensively studied member of TNF superfamily is TNF-alpha. TNF-alpha is produced in response to pathological conditions like inflammation and infection mainly by activated macrophages and T lymphocytes [4, 7], but also by several cell types including natural killer (NK) cells, mast cells, and fibroblasts. TNF-alpha is synthesized as pro-TNF, a 25 kDa plasma membrane bound protein that is further processed by metalloproteinase called TNF-alpha converting enzyme into a 17 kDa soluble form [12]. Both forms are functional in their trimeric forms via binding to their receptors. Data suggest that plasma membrane associated 25 kDa TNF-alpha form binds to the TNFR2 with high affinity whereas soluble 17 kDa form interacts with TNFR1 with high specificity [13, 14].

**Figure 1:**
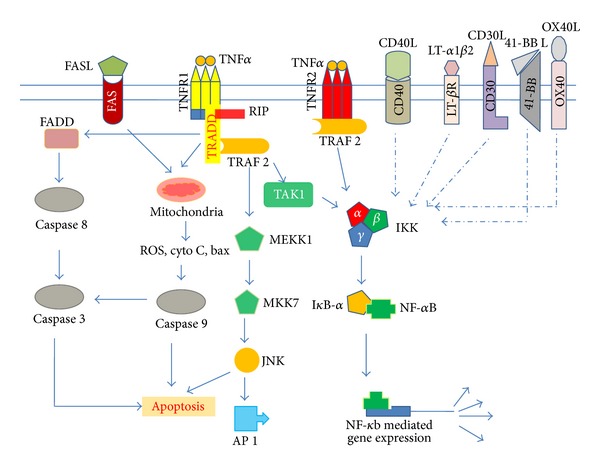
TNF and TNFR superfamily-mediated cell signaling. Binding of TNF-alpha to TNFRs results in activation of its receptors followed by recruitment of adaptor proteins (TRADD, FADD, TRAF, and RIP) in sequential manner that activates several signaling cascades leading to the activation of transcription factors NF-kappaB, AP-1, and/or caspase cascades. Most of the members of TNF superfamily activate also NF-kappaB.

TNF-alpha triggers several signaling cascades which include apoptotic pathways, NF-kappaB stimulation, and activation of p38 MAPK, ERK, and JNK [4, 7] (Figure 1). Binding of the ligand TNF-alpha to its receptor TNFR1 leads to the recruitment of a 34 kDa adapter protein called TNFR-associated death domain (TRADD). Latter interacts with the cytopathic death domain of TNFR1 through its own death domain [15] (Figure 1). Further TRADD directly binds to Fas-associated death domain protein (FADD) and activates apoptosis via caspase cascade [16] (Figure 1). On the other hand, TRADD also interacts with TNF receptor associated factor (TRAF) protein 2 followed by sequential recruitment of receptor interacting protein (RIP), TGF-beta-activated kinase 1 (TAK1), and I*κ*B kinase (IKK) complex [7, 17, 18]. IKK phosphorylates IkB resulting in the degradation of IkB. As a result, NF-kappaB translocates into the nucleus to activate transcription of effector molecules including mediators of inflammation such as chemokines, interleukin-(IL)-6, IL-8, IL-18, and cyclooxygensase-2 [4, 7, 17, 19] (Figure 1).

In addition, TNF-alpha can induce cell proliferation through induction of transcription factor called activator protein-1 (AP-1) by binding to TNFR1 followed by sequential contribution of TRADD, TRAF2-RIP, MEKK1, MKK7 and JNK [4, 7, 20] (Figure 1).

Cell signaling associated with TNFR2 is poorly understood. TNFR2 lacks death domain, despite of that it interacts with TRAF2 through which it can activate transcription factors NF-kappaB and AP-1 (Figure 1). There are several reports where TNFR2 has been reported to be involved in cellular proliferation, apoptosis, and the induction of granulocyte-macrophage colony-stimulating factor (GM-CSF) secretion [21–23].

Significance of TNF-alpha can be evaluated by this fact that several human pathogens have evolved mechanisms to combat TNF-alpha-mediated response against infection [24]. 2013 has been marked as 30 years of discovery of HIV. In this review, we will focus on TNF and TNFR and their family members in context to HIV infection and potentially how to modulate them by TNF inhibitor therapy.

## 3. Role of TNF and TNFR Superfamily Members in HIV Pathogenesis

### 3.1. TNFR1 and TNFR2

#### 3.1.1. TNF and HIV Entry

The first and foremost part of any virus life cycle is its entry into permissive cells. TNF-alpha is known to target HIV entry step specifically in macrophages but not in peripheral blood lymphocytes [25]. Notably, in cell culture, TNF-alpha is released by primary macrophages infected with HIV type 1 (HIV-1) or treated with HIV envelope protein gp120 [26]. One of the plausible strategies of inhibition of HIV entry by TNF-alpha may be by downregulating the expression of HIV receptor and coreceptors on cell surface (CD4 and CCR5) that may explain inhibition of HIV entry into permissive cells [27]. In addition, GM-CSF secretion is stimulated by TNF-alpha that in turn can downregulate CCR5 and may inhibit the entry of CCR5-dependent viruses into macrophages [28]. Moreover, it has been reported by Herbein and colleagues that pretreatment of tissue culture-differentiated macrophages (TCDM) with human recombinant TNF-alpha (hrTNF-alpha) resulted in remarkable delay in detection of HIV DNA long terminal repeat (LTR) as a result of strong inhibition of virus entry into these cells. Furthermore, using TNF-R1 and TNF-R2 mutants, they demonstrated that this inhibition was mediated through TNF-R2 not TNF-R1 [25–27].

#### 3.1.2. TNF and HIV Postentry Stages

TNF-alpha can activate HIV-1 in chronically infected T cell lines and promonocytic cell lines through translocation of NF-kappaB to the nucleus followed by activation of HIV LTR [29–33]. However, contradictory findings have been also reported where TNF-alpha has shown to inhibit HIV-1 replication in several cell types including freshly infected peripheral blood monocytes, alveolar macrophages, and TCDM [25, 34, 35]. These findings indicate that TNF-alpha may have contrasting impact on HIV-1 replication in chronically infected cells and cells coming in contact with the virus for the first time [35]. In addition, TNF-alpha induces several HIV suppressive factors such as RANTES in lymphoid cells [36, 37] and alveolar macrophages [35], MIP-1alpha, and MIP-1beta in human fetal microglia cells [38, 39] that may explain the negative role of TNF-alpha in HIV replication.

#### 3.1.3. TNF and HIV-Induced Apoptosis and Transformation

CD8+ T-cell apoptosis that occurs in HIV pathogenesis could result from the interaction between macrophage-membrane bound TNF-alpha with TNFR2 present on CD8+ T cells [40]. Additionally, HIV-1 Tat is known to induce the expression of TNF-beta in a human B-lymphoblastoid cell line (Raji cells). There is a possibility that HIV-1 Tat protein induces the growth of Kaposi's sarcoma cells via TNF-beta induction [41–43].

### 3.2. CD40

CD40 a member of TNFR superfamily, is a 45–50 kDa integral membrane glycoprotein found on B-cells, monocytes, dendritic cells, endothelial cells, and epithelial cells [44, 45]. Ligand for CD40 is CD40L (CD154), a 33 kDa transmembrane glycoprotein that is mainly expressed by activated B-cells, T-cells, and platelets [45, 46] (Figure 1). CD40-CD40L mediated signaling plays indispensable role in the development of cellular and humoral responses [46]. Membrane dissociated truncated soluble CD40L (sCD40L) is released by activated cells and binds to the CD40 molecule expressed on the target cells to activate it [46, 47].

Interestingly, increased levels of sCD40L in the cerebrospinal fluid and plasma of HIV-infected patients with cognitive impairment have been documented [46, 48]. *In vitro* experiments with recombinant CD40L (rCD40L) and HIV-1 Tat show that they act synergistically to enhance the yield of TNF-alpha by microglia and monocytes [46]. This enhancement may be contributed by ability of Tat to increase CD40 expression via NF-kappaB activation [46, 49]. Furthermore sCD40L interacts with CD40 leading to CD40-mediated signal cascade resulting in activation of NF-kappaB in microglia and monocytes [46]. This results in production of high amounts of inflammation mediators such as TNF-alpha that may explain HIV-associated dementia [46].

Tat is a multifunctional HIV protein. Notably, platelet activation has been documented in HIV-1-infected individuals [50–52]. Tat has been reported to activate platelet to release CD40L via interacting with chemokine receptor CCR3 and beta3 integrin both *in vitro *and *in vivo* [52]. Tat-stimulated CD40L activates B-cells resulting in increase in Ig yield [52]. Thus there is fair possibility that Tat-stimulated CD40L in platelet may contribute to HIV-related thrombocytopenia [52]. In addition, Tat is known to enhance the expression of TNF ligand superfamily members FasL and TRAIL in macrophages and T cells. As a consequence apoptosis can be induced in the bystander cells [53–55].

Worth mentioning, CD40L can be embedded on the surface of HIV-1 virion generated by peripheral blood and through budding from stimulated CD4+T cells in cell culture as well as in HIV-1 infected patients [56–58]. CD40L-associated virions can induce strong activation of B-cells and modulate genes including members of TNF superfamily (FAS, A20, TNIP1, CD40, lymphotoxin alpha, and lymphotoxin beta), cytokines, and transcription factors [57, 58]. Additionally, macrophages expressing Nef or activated by CD40L-CD40 receptor interaction release factors (CD23 and soluble ICAM) which makes T cells present in their vicinity susceptible to HIV infection, thus expanding the HIV cellular reservoir [59].

### 3.3. Lymphotoxin-Beta Receptor (LT-betaR)

Another important member of growing TNFR superfamily is lymphotoxin-beta receptor (LT-betaR) that governs the signaling pathways involved in the organogenesis of lymphoid tissue and function of follicular dendritic cells in a manner distinct from TNFR signaling pathways through activation of NF-kappaB [60–65] (Figure 1). LT-betaR stimulation favors HIV-1 replication in monocytes [62]. Additionally, when TNF receptors and LT-betaR are activated by their respective ligands (TNF-alpha and LT-alpha1beta2), an additive effect on HIV-1 replication is observed in U1 cells [62].

### 3.4. CD27

CD27, a TNFR superfamily member, is a 55 kDa type 1 transmembrane protein that exists in a homodimeric form [66]. Ligand for CD27 is CD27L (CD70), a member of TNF ligand superfamily. CD27 plays an important role in the activation of T cells and infection of T cells by HIV-1 [67, 68]. One critical feature of HIV-1 life cycle is its integration into the host genome using virus-encoded integrase. There are reports describing preference of HIV integration into transcriptionally active genes [68, 69]. A recent study shows that HIV-1 integrates into the coding region of CD27 gene in CD4+ T cells which may disturb CD27 open reading frame and hence can hamper the help response of CD4+ T cells [68]. This can result in inefficient removal of HIV-1 from CD4+ T cells by cytotoxic CD8+ T cells [68].

### 3.5. CD30

TNFR superfamily member CD30 is present on activated T cells, B-cells, several other transformed lymphocytes, and NK cells [70–72]. CD30 plays role in triggering developmental process in B-cells via CD30-CD30 ligand interaction [72, 73]. Ligation of CD30 with an anti-CD30 monoclonal antibody (functionally equivalent of CD30L) in chronically HIV-1-infected human T cell line ACH-2 has been reported to enhance the HIV gene expression via binding of activated NF-kappaB to the HIV-1 LTR [74, 75]. Investigation of molecular mechanism responsible for the induction of NF-kappaB revealed that NF-kappaB translocation into nucleus was mediated by TRAF2, independent of TNF-alpha/beta (Figure 1).

### 3.6. Fas

The Fas also known as Apo-1, CD95 and TNFSF6, is a TNFR superfamily member that governs apoptosis when activated by its ligand FasL (Figure 1). Role of Fas-FasL signaling cascade in HIV pathogenesis has been extensively reviewed [55, 76]. Production of Fas and FasL is increased in CD4+ T cells isolated from HIV infected individuals [77, 78]. Increased expression of Fas is observed in B-cells, CD4+, and CD8+ T cells whereas FasL increased expression is associated with macrophages, NK cells, and monocytes [78, 79]. *In vitro* studies reveal that HIV-infected macrophages can induce apoptosis in Jurkat T cells and in peripheral blood T lymphocytes via FasL that could be one factor responsible for the depletion of lymphocytes during HIV pathogenesis [78, 80].

### 3.7. 4-1BB (CD137)

4-1BB (CD137) is a TNFR expressed predominately on T cells, NK cells, mast cells, and neutrophils [4]. Ligation of 4-1BB with agonistic monoclonal antibodies has been shown to effectively increase the HIV-1 replication in CD4+ T cells isolated from HIV-1 infected patients. There is a possibility that 4-1BB receptor may be involved in the activation of HIV from latency in CD4+ T cells [81].

### 3.8. OX40 (CD134)

OX40 (CD134), a member of the TNFR superfamily, is expressed on activated CD4+ T cells and neutrophils. It is crucial for the survival of antigen specific CD4+ T cells [4, 82]. Natural ligand for this receptor is OX40L (also called CD252 and gp34), a member of TNFL superfamily. OX40-gp34 interaction, in HIV-1 (both acutely and chronically) infected T cell lines result in increase in HIV-1 replication, independent of TNF-alpha or TNF-beta production. The increase in viral replication has been shown to mediate by activation of NF-kappaB followed by stimulation of HIV-1 LTR [33]. Recent study suggests that OX40 activation suppresses the CCR5-tropic (R5) HIV-1 infection in PBMCs by generating anti-HIV beta-chemokines [83].

### 3.9. DR 4 and DR 5

Death receptor (DR), DR4 (also called TRAILR1, TNFRSF10A, and Apo2) and DR5 (also called TRAILR2 and TNFRSF10B) are the fourth and fifth members of TNFR superfamily, respectively. They are expressed in most of the normal as well as transformed cells [4]. They govern their activity by binding to their ligand TRAIL (tumor-necrosis-factor related apoptosis inducing ligand or Apo2L) leading to receptor oligomerization, assembly of death inducing signaling complexes that ultimately govern the activation of caspase pathways [4, 78]. TRAIL is a TNFL superfamily member expressed in NK, T cells, and dendritic cells. In HIV-infected CD4+ T cells, TRAIL is involved in the apoptosis of infected cells by binding with DR4 and DR5 [4, 78]. Plasma level of TRAIL has been found to be high in HIV-1 infected individuals, whereas HIV-1 infected patients undergoing antiretroviral therapy demonstrate decrease in TRAIL levels in plasma and also decreased viral load suggesting crucial role of TRAIL in HIV-1 pathogenesis [84]. *In vitro* testing of recombinant TRAIL (rTRAIL) against HIV-infected peripheral blood lymphocytes and monocyte-derived macrophages isolated from HIV-infected patients results in apoptosis of the target cells. However, rTRAIL shows no effect against target cells isolated from uninfected patients [84]. This raises the possibility of using TRAIL as anti-HIV agent.

## 4. HIV Proteins Mimicking TNF/TNFR Signaling

**Figure 2:**
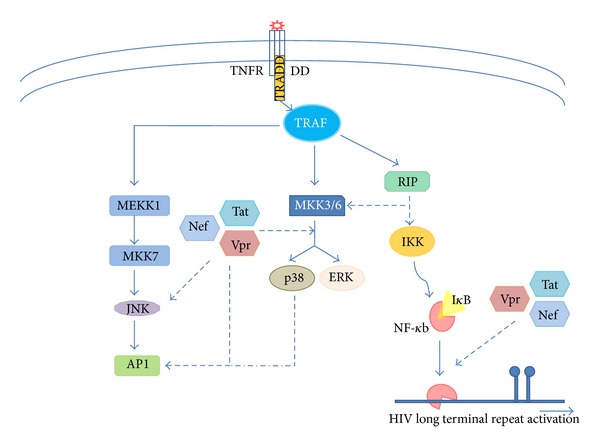
TNF signaling mimicking by HIV proteins. Early expressed HIV proteins Vpr, Tat, and Nef mimic TNF-alpha as they activate TNF-alpha governing signaling pathways to enhance HIV replication in infected cells.

Several HIV proteins especially Vpr, Tat, and Nef exhibit molecular mimicry with respect to TNF signaling in HIV-infected cells particularly in macrophages (Figure 2).

Viral protein R (Vpr) is a small (14 kDa) multifunctional virion-associated accessory protein that participates in import of viral preintegration complexes to the nucleus. In addition, Vpr exerts antiapoptotic effect in HIV infected cells on the other hand, it induces apoptosis in the surrounding cells [85]. Besides these functions, Vpr also triggers mitochondrial dysfunction [86, 87]. Although Vpr is dispensable for HIV replication in T cells, it is critical for HIV replication in nondividing cells, for example, macrophages [10] (Figure 2). There are several reports where Vpr has been shown to stimulate HIV-1 growth using serum derived or synthetic Vpr [88, 89]. Virion derived (HIV-1) Vpr activates NF-kappaB in primary T cells, macrophages as well as in promonocytic cell line U937 [90]. Similarly, synthetic Vpr has been shown to activate NF-kappaB, AP-1, and JNK in primary macrophages and U937 cells resulting in increase in viral replication [10, 89] (Figure 2). In addition, recombinant Vpr stimulates HIV production by utilizing toll-like receptor 4 (TLR4) and IL-6 secretion [87]. Synergistic effect of Vpr and Tat leads to enhanced stimulation of HIV-1 LTR [91]. Even Tat alone mimics TNF-alpha. Like TNF-alpha, Tat triggers translocation of NF-kappaB into nucleus and activation of AP-1/cJun by MAPK activation JNK, p38, and ERK1/2 [10, 92] (Figure 2).

In contrast, there are several reports describing the suppression of NF-kappaB activity by Vpr. It has been shown that glucocorticoid receptor and Vpr act in harmony to inhibit NF-kappaB mediated gene expression via a pathway involving the suppression of poly (ADP-ribose) polymerase (PARP)-1 nuclear trafficking in response to TNF-alpha [85, 93].

HIV Nef, the most abundantly expressed HIV accessory protein, is a multifunctional 27 kDa myristoylated cytoplasmic protein expressed in early phase of viral life cycle [94]. Nef helps in the establishment of HIV persistence in infected cells [95] and interferes with several signaling events [10]. Recombinant Nef (rNef) has been shown to induce expression and release of several cytokines mediated by NF-kappaB activation in culture monocyte-derived macrophages (MDMs) [96, 97]. In U937 cells and MDMs, exogenously added rNef triggers NF-kappaB activation resulting in HIV LTR activation [98] (Figure 2). In addition, rNef induce, transcription of several inflammatory genes in response to addition of rNef to MDMs. Convincible, analysis of rNef treated MDMs supernatants revealed induction and release of TNF-alpha and other macrophage inflammatory proteins (MIP-1alpha and MIP-1beta) and IL6 [96]. Moreover, in chronically infected promonocytic cells U1, addition of rNef leads to increase in HIV-1 replication [98]. Furthermore, rNef is able to rapidly and transiently induce phosphorylation of several key-signaling molecules including alpha/beta subunits of Ikappa B kinase, ERK1/2, JNK, and p38 in MDMs [97]. Signaling scenario observed in post-rNef treatment is more or less similar to what is observed in post TNF-alpha treatment [10] suggesting their similar impact on HIV infection at least in mononuclear macrophages (Figure 2).

## 5. Targeting Members of TNF and TNFR Superfamily in HIV-1 Infection

In this section, we will discuss current status and future potential therapeutic use of TNF based therapies for HIV-1 and HIV-1 related diseases and their pros and cons.

The targeting of TNF signaling has proven the most successful and clinical efficacious therapy at reducing the inflammation in several diseases. Several TNF related inflammatory cytokines and their cognate receptors are now in preclinical or clinical phase of development as a possible target for treating various diseases such as cancer, autoimmune, and inflammatory disorders. HIV infection is characterized by immune activation and inflammation [99]. Therefore, TNF blocking agents and/or TNF inhibitor therapy could be useful to modulate HIV disease.

There are several types of drugs targeting TNF and TNFR superfamily that are being used for therapeutic application [100]. Five different antibodies or receptors based drugs targeting TNF and LTalpha are approved for treating various inflammatory diseases. The chimeric antibody infliximab, TNF targeted drug, was approved in 1998 followed by etanercept [101]. The fully human antibodies adalimumab and golimumab were approved in 2002 and 2009, respectively. Certolizumab pegol, an another therapeutic monoclonal antibody with pegylated Fab fragment was approved in 2008. As polyethylene glycol does not cross the placenta, this drug can be administrated to pregnant women who have autoimmune diseases and are in need of anti-TNF-alpha therapy [102]. These drugs neutralize the biological activity of TNF-alpha by binding with high affinity to the soluble as well as transmembrane form of TNF-alpha. Thus they prevent the binding of TNF-alpha to their natural receptors. Worth mentioning, drugs adalimumab and infliximab have the potential to lyse the cells involved in inflammation. Besides the availability and affordability, these drugs fall in the class of immune suppressors which may have serious complications such as blood disorders, infections, liver injury, skin lesion, and reactivation of tuberculosis [100].

### 5.1. Anti-TNF Therapy and HIV-1 Infection

HIV-1 infection induces TNF expression, and high amount of TNF is present in all stages of HIV-1 infection [29, 103, 104]. This elevated level of serum TNF has been associated with increased viral replication and depletion of CD4+ T cells [105, 106]. The treatment of HIV-1 infected patients with thalidomide (a weak TNF inhibitor) reduces serum TNF level that results in lower viral load [107, 108]. Furthermore, it has been shown that LMP-420, a small inhibitor of TNF, suppresses the transcription and biosynthesis of TNF, which ultimately inhibits the replication of HIV-1 [109]. Multiple studies have reported that use of anti-TNF therapy in patients with HIV-1 does not appear to increase the mortality rates [110]. As there is a theoretical risk that immunosuppressive drugs increase the risk of opportunistic infection and progression to HIV-1 disease, several studies have shown that anti-TNF therapy may improve the HIV-1 associated symptoms [111]. Further, etanercept has been used as anti-TNF therapy in HIV-1 infected patients for the treatment of rheumatic disease. In most of the cases, the therapy was well tolerated. In addition, no opportunistic infection was observed in HIV-1 infected rheumatic patients unless they had uncontrolled HIV [112, 113]. Similar results were obtained by using other anti-TNF agents such as infliximab and adalimumab in HIV-1 infected patients. These drugs were safe and effective with normal CD4+ T cell counts [114, 115]. Therefore, in the patients whose HIV disease is under control of HAART, the anti-TNF therapy may be helpful for the treatment of autoimmune disease without enhancing plasma viremia [111, 116]. Worth mentioning, above clinical studies are based on a little number of patients; therefore, results must be analyzed based on large cohorts to assert a safe and real benefit to the community.

### 5.2. Costimulatory TNFRs and HIV-1-Specific T Cell Response

**Figure 3:**
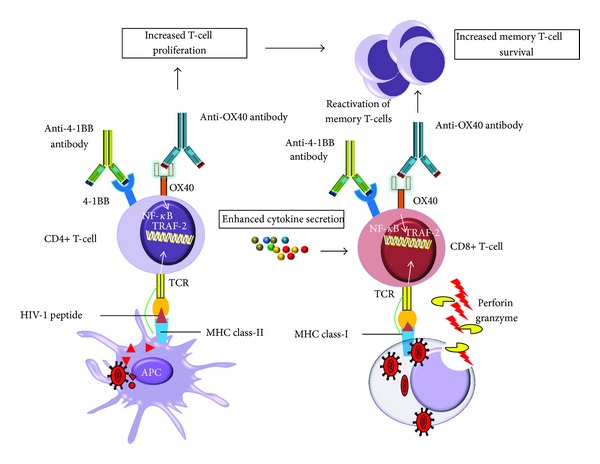
4-1BB and OX40 signaling enhance anti-HIV immunity, leading to therapeutic effects. Agonists 4-1BB or OX40 specific antibodies can induce enhanced anti-HIV T cell (CD4+ and CD8+ T cell) response in HIV-1 infected patients. The ligation of OX40/4-1BB antibodies activates OX40/4-1BB signaling that results in enhanced CD4+ T cell response through increased cytokine production and increased survival of memory T cells. TRAFs and NF-kappaB signaling has been shown to be important for the generation of memory T cells. Potential antiviral mechanisms of OX40/4-1BB include an increase in CD8+ T cell cytotoxicity through perforins and granzymes and an increase in Fas/FasL mediated HIV-infected cell killing.

Several studies have been carried out to compare the efficacy of different costimulatory TNFR family members for the activation of HIV-1-specific T cells *in vitro*. These costimulatory signaling pathways could be used to activate CD8 T cell responses to HIV *in vivo *(Figure 3) [117]. The OX40L signaling pathway plays an important costimulatory role in DC/T cell interactions. OX40 binding to CD4+ T cells by human OX40L-IgG1 enhances *ex vivo* expansion of HIV-1 specific CTL from HIV-1 infected individuals [118]. This mechanism of CTL expansion was independent of induction of cytokines such as IL-2 or any inhibitory effect on CD4+ T helper cells, but it was associated with a direct effect on proliferation of CD4+ T cells. This mechanism of action of OX40 represents a potentially novel immunotherapeutic strategy that could prevent the persistent HIV-1 infection [118]. Like OX40, the 4-1BB is transiently expressed following TCR ligation [119, 120]. Furthermore, the ligation of 4-1BB/4-1BBL enhances CTL expansion that has both antiviral and antitumor activity [121]. In addition, dual costimulation by OX40L in combination with 4-1BBL resulted in improved expansion and effector function of CTL over costimulation with individual costimulatory molecules [122]. Furthermore, urelumab is a monoclonal antibody that specifically binds to and activates 4-1BB expressing immune cells and stimulates the CTL response against tumor cells. Although 4-1BB ligand (4-1BBL) could be an important costimulatory molecule for exhausted CD8+ T cells from chronically infected patients, anti-4-1BB therapy is associated with several immunological side effects such as splenomegaly, hepatitis, and several immunological disorders [123, 124]. Additionally, it has been reported that stimulation of 4-1BB in T cells enhances HIV-1 replication [81]. To be an effective therapy, the 4-1BB agonist should induce HIV-1 specific CD8+ T cell response and also should not induce viral replication. This issue may be solved by incorporation of 4-1BBL along with CD8+ T cell epitopes into vaccine vectors. The incorporation of 4-1BBL into a fowlpox vector along with HIV-1 Gag enhances Gag-specific CD8+ T cell responses, suggesting this could be a useful approach in a therapeutic vaccine [125].

### 5.3. TNF and TNFR Superfamily and HIV-1 Reservoirs

**Figure 4:**
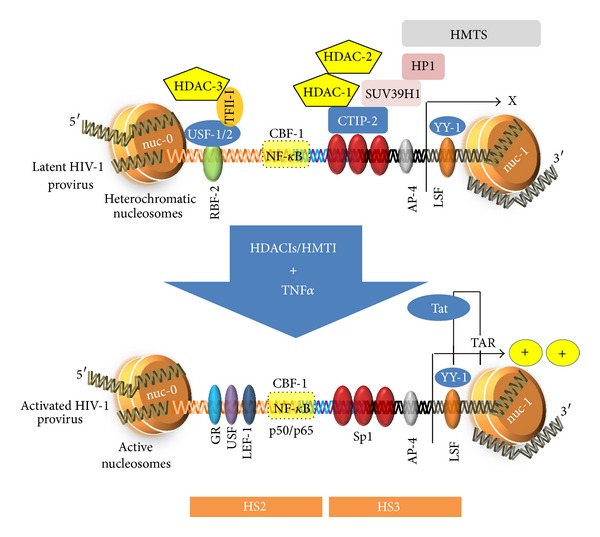
Potential TNF-based therapies to reactivate HIV-1 from latency. During HIV-1 latency, nuc-1 nucleosome is hypoacetylated. CTIP-2 interacts with Sp1, switching nuc-1 from transcriptionally active to a repressive state. Furthermore, CTIP-2 recruits HDACs that deacetylate the nuc-1 nucleosome. SUV39H1 adds trimethylation mark onto the histone protein H3. Furthermore, HP1 protein stabilizes the nuc-1 in transcriptionally silent state. The combination of TNF-alpha with HDACIs or HMTIs can disrupt the HIV-1 latency. TNF-alpha and HDACIs can trigger the activation of transcriptional activators, such as NF-kappaB (p50/p65 heterodimer). HDACIs may prevent the formation of heterochromatin, resulting in nuc-1 hyperacetylation and remodeling, thereby alleviating the HIV transcriptional block. The use of TNF, HDACIs, and HMTIs enhances HIV-1 LTR transcription and could participate in the clearance of HIV-1 from cellular reservoirs and ultimately could lead to the cure of HIV-infected patients.

The persistence of HIV-1 reservoirs is a major challenge for complete viral eradication in HIV-1 infected patients [126–128]. To date, resting or memory CD4+ T cells are the most well-characterized HIV-1 reservoirs [128]. During the development of memory CD4+ T cells, the NF-kappaB signaling ensures T cell survival during the initial differentiation of effector cells into memory T cells. OX40 along with CD30 induces NF-kappaB-dependent expression of antiapoptotic genes such as Bcl-2 and Bcl-xL that in turn play an important role in the survival of memory CD4+ T cells within the small intestine lamina propria [129]. Low levels of NF-kappaB activity by control of the TNF and TNFR superfamily members may contribute to the establishment and maintenance of latent HIV-1 reservoirs in memory CD4+ T cells. Activation induced purging of HIV-1 in patients receiving HAART has long been a proposed mechanism to eradicate the latent HIV pool [130]. TNF-alpha has been used to reactivate HIV-1 from latently infected cells, but it is not the optimal reactivation treatment [131]. In Jurkat based HIV-1 latency model, TNF-alpha consistently activates latent HIV-1 provirus. However, in primary CD4 T cell model, TNF-alpha does not appear too effective to purge HIV-1 from latent reservoirs [132, 133]. Moreover, there are some concerns about the toxicity associated with TNF treatment [78]. Since TNF-alpha is only a weak enhancer of HIV-1 reactivation to purge cellular reservoirs, new approaches have been used to target the epigenetic regulation of HIV gene expression (Figure 4). Many studies have reported that the combination of HDACIs (histone deacetylase inhibitors) with TNF-alpha synergistically reactivates HIV-1 from latency [134, 135]. The HDACIs such as trichostatin A (TSA), trapoxin (TPX), valproic acid (VPA), and sodium butyrate (NaBut) activate HIV-1 transcription by remodeling nuc-1 of HIV-1 promoter [136, 137]. Thus, an ideal anti-AIDS therapy would consist in eliminating the pool of latently cells by inducing forced HIV-1 gene expression by HDACIs and TNF-alpha, while maintaining an effective HAART regimen [131, 136, 138]. Furthermore, HDACIs potentiate TNF-alpha mediated NF-kappaB activation and also delay I*κ*Balpha cytoplasmic reappearance [139]. Thus, the use of TNF-alpha and HDACIs in the presence of HAART not only could purge HIV-1 for latent reservoirs but also could suppress plasma viremia and formation of further new viral reservoirs. In addition to HDACI, inhibitors of histone methyltransferase (HMTI) have also been recently shown to reactivate HIV-1 from latency in resting CD4+ T cells from HIV-infected HAART-treated patients [140]. Furthermore, JNK inhibitors, such as AS601245, prevent HIV-1 reactivation from latency despite potent NF-kappaB activity [141]. Since stimulation of CD40 and 4-1BB induces JNK activation [142], the use of agonist of 4-1BB or CD40 could lead to the reactivation of the HIV-1 from latency. Combining TNF-alpha treatment with HDACIs/HMTI could have significant impact on the clearance of HIV-1 from cellular reservoirs and ultimately could lead to the cure of HIV-infected patients [143].

## 6. Conclusion

TNF ligands and TNF receptors superfamily are the integral part of our immune system. TNF signaling exerts significant impact on HIV life cycle. In turn, HIV encoded proteins also modulate TNF signaling pathways resulting in the survival of HIV-infected cells and killing of bystander cells. Anti-TNF therapy has been successfully used in several inflammatory diseases. Combinatorial therapy involving HAART, anti-TNF therapy, along with use of HDACIs/HMTI might be a viable option for the treatment of HIV infection to reach the ultimate goal, the clearance of HIV-1 from cellular reservoirs.

## References

[1] Carswell EA, Old LJ, Kassel RL (1975). An endotoxin induced serum factor that causes necrosis of tumors. *Proceedings of the National Academy of Sciences of the United States of America*.

[2] Beutler B, Cerami A (1986). Cachectin and tumour necrosis factor as two sides of the same biological coin. *Nature*.

[3] Bradley JR (2008). TNF-mediated inflammatory disease. *Journal of Pathology*.

[4] Aggarwal BB, Gupta SC, Kim JH (2012). Historical perspectives on tumor necrosis factor and its superfamily: 25 years later, a golden journey. *Blood*.

[5] Moelants EA, Mortier A, van Damme J, Proost P (2013). Regulation of TNF-alpha with a focus on rheumatoid arthritis. *Immunology & Cell Biology*.

[6] Aggarwal BB (2003). Signalling pathways of the TNF superfamily: a double-edged sword. *Nature Reviews Immunology*.

[7] Herbein G, O’Brien WA (2000). Tumor necrosis factor (TNF)-*α* and TNF receptors in viral pathogenesis. *Proceedings of the Society for Experimental Biology and Medicine*.

[8] Pennica D, Nedwin GE, Hayflick JS (1984). Human tumour necrosis factor: precursor structure, expression and homology to lymphotoxin. *Nature*.

[9] Kelker HC, Oppenhaim JD, Stone-Wolff D (1985). Characterization of human tumor necrosis factor produced by peripheral blood monocytes and its separation from lymphotoxin. *International Journal of Cancer*.

[10] Herbein G, Khan KA (2008). Is HIV infection a TNF receptor signalling-driven disease?. *Trends in Immunology*.

[11] Beutler B, van Huffel C (1994). Unraveling function in the TNF ligand and receptor families. *Science*.

[12] Black RA, Rauch CT, Kozlosky CJ (1997). A metalloproteinase disintegrin that releases tumour-necrosis factor-*∅* from cells. *Nature*.

[13] Grell M, Wajant H, Zimmermann G, Scheurich P (1998). The type 1 receptor (CD120a) is the high-affinity receptor for soluble tumor necrosis factor. *Proceedings of the National Academy of Sciences of the United States of America*.

[14] Grell M, Douni E, Wajant H (1995). The transmembrane form of tumor necrosis factor is the prime activating ligand of the 80 kDa tumor necrosis factor receptor. *Cell*.

[15] Hsu H, Xiong J, Goeddel DV (1995). The TNF receptor 1-associated protein TRADD signals cell death and NF-*κ*B activation. *Cell*.

[16] Hsu H, Shu H, Pan M, Goeddel DV (1996). TRADD-TRAF2 and TRADD-FADD interactions define two distinct TNF receptor 1 signal transduction pathways. *Cell*.

[17] DiDonato JA, Hayakawa M, Rothwarf DM, Zandi E, Karin M (1997). A cytokine-responsive IkappaB kinase that activates the transcription factor NF-kappaB. *Nature*.

[18] Mercurio F, Zhu H, Murray BW (1997). IKK-1 and IKK-2: cytokine-activated I*κ*B kinases essential for NF-*κ*B activation. *Science*.

[19] Devin A, Cook A, Lin Y, Rodriguez Y, Kelliher M, Liu Z (2000). The distinct roles of TRAF2 and RIP in IKK activation by TNF-R1: tRAF2 recruits IKK to TNF-R1 while RIP mediates IKK activation. *Immunity*.

[20] Natoli G, Costanzo A, Moretti F, Fulco M, Balsano C, Levrero M (1997). Tumor necrosis factor (TNF) receptor 1 signaling downstream of TNF receptor-associated factor 2. Nuclear factor *κ*B (NF*κ*B)-inducing kinase requirement for activation of activating protein 1 and NF*κ*B but not of c-Jun N-terminal kinase/stress-activated protein kinase. *The Journal of Biological Chemistry*.

[21] Grell M, Becke FM, Wajant H, Mنnnel DN, Scheurich P (1998). TNF receptor type 2 mediates thymocyte proliferation independently of TNF receptor type 1. *European Journal of Immunology*.

[22] Gehr G, Gentz R, Brockhaus M, Loetscher H, Lesslauer W (1992). Both tumor necrosis factor receptor types mediate proliferative signals in human mononuclear cell activation. *Journal of Immunology*.

[23] Zheng L, Fisher O, Miller RE, Peschon J, Lynch DH, Lenardo MJ (1995). Induction of apoptosis in mature T cells by tumour necrosis factor. *Nature*.

[24] Rahman MM, McFadden G (2006). Modulation of tumor necrosis factor by microbial pathogens. *PLoS Pathogens*.

[25] Herbein G, Montaner LJ, Gordon S (1996). Tumor necrosis factor alpha inhibits entry of human immunodeficiency virus type 1 into primary human macrophages: a selective role for the 75- kilodalton receptor. *Journal of Virology*.

[26] Karsten V, Gordon S, Kirn A, Herbein G (1996). HIV-1 envelope glycoprotein gp120 down-regulates CD4 expression in primary human macrophages through induction of endogenous tumour necrosis factor-*α*. *Immunology*.

[27] Herbein G, Doyle AG, Montaner LJ, Gordon S (1995). Lipopolysaccharide (LPS) down-regulates CD4 expression in primary human macrophages through induction of endogenous tumour necrosis factor (TNF) and IL-1*β*. *Clinical and Experimental Immunology*.

[28] Di Marzio P, Tse J, Landau NR (1998). Chemokine receptor regulation and HIV type 1 tropism in monocyte-macrophages. *AIDS Research and Human Retroviruses*.

[29] Duh EJ, Maury WJ, Folks TM, Fauci AS, Rabson AB (1989). Tumor necrosis factor *α* activates human immunodeficiency virus type 1 through induction of nuclear factor binding to the NF-*κ*B sites in the long terminal repeat. *Proceedings of the National Academy of Sciences of the United States of America*.

[30] Griffin GE, Leung K, Folks TM, Kunkel S, Nabel GJ (1989). Activation of HIV gene expression during monocyte differentiation by induction of NF-*κ*B. *Nature*.

[31] Okamoto T, Matsuyama T, Mori S (1989). Augmentation of human immunodeficiency virus type 1 gene expression by tumor necrosis factor *α*. *AIDS Research and Human Retroviruses*.

[32] Osborn L, Kunkel S, Nabel GJ (1989). Tumor necrosis factor *α* and interleukin 1 stimulate the human immunodeficiency virus enhancer by activation of the nuclear factor *κ*B. *Proceedings of the National Academy of Sciences of the United States of America*.

[33] Takahashi Y, Tanaka Y, Yamashita A, Koyanagi Y, Nakamura M, Yamamoto N (2001). OX40 stimulation by gp34/OX40 ligand enhances productive human immunodeficiency virus type 1 infection. *Journal of Virology*.

[34] Herbein G, Gordon S (1997). 55- and 75-kilodalton tumor necrosis factor receptors mediate distinct actions in regard to human immunodeficiency virus type 1 replication in primary human macrophages. *Journal of Virology*.

[35] Lane BR, Markovitz DM, Woodford NL, Rochford R, Strieter RM, Coffey MJ (1999). TNF-*α* inhibits HIV-1 replication in peripheral blood monocytes and alveolar macrophages by inducing the production of RANTES and decreasing C-C chemokine receptor 5 (CCR5) expression. *Journal of Immunology*.

[36] Coffey MJ, Woffendin C, Phare SM, Strieter RM, Markovitz DM (1997). RANTES inhibits HIV-1 replication in human peripheral blood monocytes and alveolar macrophages. *American Journal of Physiology—Lung Cellular and Molecular Physiology*.

[37] Simmons G, Clapham PR, Picard L (1997). Potent inhibition of HIV-1 infectivity in macrophages and lymphocytes by a novel CCR5 antagonist. *Science*.

[38] McManus CM, Brosnan CF, Berman JW (1998). Cytokine induction of MIP-1*α* and MIP-1*β* in human fetal microglia. *Journal of Immunology*.

[39] Kitai R, Zhao M, Zhang N, Hua LL, Lee SC (2000). Role of MIP-1*β* and RANTES in HIV-1 infection of microglia: inhibition of infection and induction by IFN*β*. *Journal of Neuroimmunology*.

[40] Herbein G, Mahlknecht U, Batliwalla F (1998). Apoptosis of CD8^+^ T cells is mediated by macrophages through interaction of HIV gp120 with chemokine receptor CXCR4. *Nature*.

[41] Sastry KJ, Reddy RHR, Pandita R, Totpal K, Aggarwal BB (1990). HIV-1 tat gene induces tumor necrosis factor-*β* (lymphotoxin) in a human B-lymphoblastoid cell line. *The Journal of Biological Chemistry*.

[42] Ensoli B, Barillari G, Salahuddin SZ, Gallo RC, Wong-Staal F (1990). Tat protein of HIV-1 stimulates growth of cells derived from Kaposi’s sarcoma lesions of AIDS patients. *Nature*.

[43] Tang Q, Qin D, Lv Z (2012). Herpes simplex virus type 2 triggers reactivation of Kaposi’s sarcoma-associated herpesvirus from latency and collaborates with HIV-1 tat. *PLoS ONE*.

[44] Graf D, Müller S, Korthäuer U, van Kooten C, Weise C, Kroczek RA (1995). A soluble form of TRAP (CD40 ligand) is rapidly released after T cell activation. *European Journal of Immunology*.

[45] van Kooten G, Banchereau J (2000). CD40-CD40 ligand. *Journal of Leukocyte Biology*.

[46] Sui Z, Sniderhan LF, Schifitto G (2007). Functional synergy between CD40 ligand and HIV-1 Tat contributes to inflammation: implications in HIV type 1 dementia. *Journal of Immunology*.

[47] Mazzei GJ, Edgerton MD, Losberger C (1995). Recombinant soluble trimeric CD40 ligand is biologically active. *The Journal of Biological Chemistry*.

[48] Sipsas NV, Sfikakis PP, Kontos A, Kordossis T (2002). Levels of soluble CD40 ligand (CD154) in serum are increased in human immunodeficiency virus type 1-infected patients and correlate with CD4^+^ T-cell counts. *Clinical and Diagnostic Laboratory Immunology*.

[49] D’Aversa TG, Eugenin EA, Berman JW (2005). NeuroAIDS: contributions of the human immunodeficiency virus-1 proteins Tat and gp120 as well as CD40 to microglial activation. *Journal of Neuroscience Research*.

[50] Ahmad R, Iannello A, Samarani S (2006). Contribution of platelet activation to plasma IL-18 concentrations in HIV-infected AIDS patients. *AIDS*.

[51] Holme PA, Müller F, Solum NO, Brosstad F, Froland SS, Aukrust P (1998). Enhanced activation of platelets with abnormal release of RANTES in human immunodeficiency virus type 1 infection. *The FASEB Journal*.

[52] Wang J, Zhang W, Nardi MA, Li Z (2011). HIV-1 Tat-induced platelet activation and release of CD154 contribute to HIV-1-associated autoimmune thrombocytopenia. *Journal of Thrombosis and Haemostasis*.

[53] Campbell GR, Pasquier E, Watkins J (2004). The glutamine-rich region of the HIV-1 Tat protein is involved in T-cell apoptosis. *The Journal of Biological Chemistry*.

[54] Zhang M, Li X, Pang X (2001). Identification of a potential HIV-induced source of bystander-mediated apoptosis in T cells: upregulation of TRAIL in primary human macrophages by HIV-1 Tat. *Journal of Biomedical Science*.

[55] Poonia B, Pauza CD, Salvato MS (2009). Role of the Fas/FasL pathway in HIV or SIV disease. *Retrovirology*.

[56] Martin G, Tremblay MJ (2004). HLA-DR, ICAM-1, CD40, CD40L, and CD86 are incorporated to a similar degree into clinical human immunodeficiency virus type 1 variants expanded in natural reservoirs such as peripheral blood mononuclear cells and human lymphoid tissue cultured ex vivo. *Clinical Immunology*.

[57] Martin G, Roy J, Barat C, Ouellet M, Gilbert C, Tremblay MJ (2007). Human immunodeficiency virus type 1-associated CD40 ligand transactivates B lymphocytes and promotes infection of CD4^+^ T cells. *Journal of Virology*.

[58] Imbeault M, Ouellet M, Giguère K (2011). Acquisition of host-derived CD40L by HIV-1 in vivo and its functional consequences in the B-cell compartment. *Journal of Virology*.

[59] Swingler S, Brichacek B, Jacque J, Ulich C, Zhou J, Stevenson M (2003). HIV-1 Nef intersects the macrophage CD40L signalling pathway to promote resting-cell infection. *Nature*.

[60] Mackay F, Majeau GR, Lawton P, Hochman PS, Browning JL (1997). Lymphotoxin but not tumor necrosis factor functions to maintain splenic architecture and humoral responsiveness in adult mice. *European Journal of Immunology*.

[61] Murphy M, Walter BN, Pike-Nobile L (1998). Expression of the lymphotoxin *β* receptor on follicular stromal cells in human lymphoid tissues. *Cell Death and Differentiation*.

[62] Marshall WL, Brinkman BMN, Ambrose CM (1999). Signaling through the lymphotoxin-*β* receptor stimulates HIV-1 replication alone and in cooperation with soluble or membrane-bound TNF-*α*. *Journal of Immunology*.

[63] Locksley RM, Killeen N, Lenardo MJ (2001). The TNF and TNF receptor superfamilies: integrating mammalian biology. *Cell*.

[64] Dejardin E, Droin NM, Delhase M (2002). The lymphotoxin-*β* receptor induces different patterns of gene expression via two NF-*κ*B pathways. *Immunity*.

[65] Wang C, Watts TH (2012). Maintaining the balance: costimulatory TNFRs and control of HIV. *Cytokine & Growth Factor Reviews*.

[66] Camerini D, Walz G, Loenen WAM, Borst J, Seed B (1991). The T cell activation antigen CD27 is a member of the nerve growth factor/tumor necrosis factor receptor gene family. *Journal of Immunology*.

[67] Libregts S, van Olffen RW, van der Sluijs KF, van Lier RAW, Nolte MA (2011). Function of CD27 in helper T cell differentiation. *Immunology Letters*.

[68] Ohmori R, Tsuruyama T (2012). In vitro HIV-1 LTR integration into T-cell activation gene CD27 segment and the decoy effect of modified-sequence DNA. *PLoS ONE*.

[69] Schröder ARW, Shinn P, Chen H, Berry C, Ecker JR, Bushman F (2002). HIV-1 integration in the human genome favors active genes and local hotspots. *Cell*.

[70] Durkop H, Latza U, Hummel M, Eitelbach F, Seed B, Stein H (1992). Molecular cloning and expression of a new member of the nerve growth factor receptor family that is characteristic for Hodgkin’s disease. *Cell*.

[71] Del Prete G, De Carli M, Almerigogna F (1995). Preferential expression of CD30 by human CD4^+^ T cells producing Th2-type cytokines. *The FASEB Journal*.

[72] Tsitsikov EN, Wright DA, Geha RS (1997). CD30 induction of human immunodeficiency virus gene transcription is mediated by TRAF2. *Proceedings of the National Academy of Sciences of the United States of America*.

[73] Shanebeck KD, Maliszewski CR, Kennedy MK (1995). Regulation of murine B cell growth and differentiation by CD30 ligand. *European Journal of Immunology*.

[74] Biswas P, Smith CA, Goletti D, Hardy EC, Jackson RW, Fauci AS (1995). Cross-linking of CD30 induces HIV expression in chronically infected T cells. *Immunity*.

[75] Maggi E, Annunziato F, Manetti R (1995). Activation of HIV expression by CD30 triggering in CD4^+^ cells from HIV-infected individuals. *Immunity*.

[76] Dianzani U, Bensi T, Savarino A (2003). Role of FAS in HIV infection. *Current HIV Research*.

[77] Mitra D (1996). HIV-1 upregulates Fas ligand expression in CD4^+^ T cells in vitro and in vivo: association with Fas-mediated apoptosis and modulation by aurintricarboxylic acid. *Immunology*.

[78] Cummins NW, Badley AD (2010). Mechanisms of HIV-associated lymphocyte apoptosis: 2010. *Cell Death and Disease*.

[79] Kottilil S, Jackson JO, Reitano KN (2007). Innate immunity in HIV infection: enhanced susceptibility to CD95-mediated natural killer cell death and turnover induced by HIV viremia. *Journal of Acquired Immune Deficiency Syndromes*.

[80] Badley AD, McElhinny JA, Leibson PJ, Lynch DH, Alderson MR, Paya CV (1996). Upregulation of fas ligand expression by human immunodeficiency virus in human macrophages mediates apoptosis of uninfected T lymphocytes. *Journal of Virology*.

[81] Wang S, Kim Y, Bick C, Kim SH, Kwon BS (1998). The potential roles of 4-1BB costimulation in HIV type 1 infection. *AIDS Research and Human Retroviruses*.

[82] Takahashi Y, Tanaka R, Yamamoto N, Tanaka Y (2008). Enhancement of OX40-induced apoptosis by TNF coactivation in OX40-expressing T cell lines in vitro leading to decreased targets for HIV type 1 production. *AIDS Research and Human Retroviruses*.

[83] Tanaka R, Takahashi Y, Kodama A, Saito M, Ansari AA, Tanaka Y (2010). Suppression of CCR5-tropic HIV type 1 infection by OX40 stimulation via enhanced production of *β*-chemokines. *AIDS Research and Human Retroviruses*.

[84] Herbeuval J, Boasso A, Grivel J (2005). TNF-related apoptosis-inducing ligand (TRAIL) in HIV-1-infected patients and its in vitro production by antigen-presenting cells. *Blood*.

[85] Kogan M, Deshmane S, Sawaya BE, Gracely EJ, Khalili K, Rappaport J (2013). Inhibition of NF-kappaB activity by HIV-1 Vpr is dependent on Vpr binding protein. *Journal of Cellular Physiology*.

[86] Malim MH, Emerman M (2008). HIV-1 accessory proteins—ensuring viral survival in a hostile environment. *Cell Host and Microbe*.

[87] Hoshino S, Konishi M, Mori M (2010). HIV-1 Vpr induces TLR4/MyD88-mediated IL-6 production and reactivates viral production from latency. *Journal of Leukocyte Biology*.

[88] Levy DN, Refaeli Y, Macgregor RR, Weiner DB (1994). Serum Vpr regulates productive infection and latency of human immunodeficiency virus type 1. *Proceedings of the National Academy of Sciences of the United States of America*.

[89] Varin A, Decrion A, Sabbah E (2005). Synthetic Vpr protein activates activator protein-1, c-Jun N-terminal kinase, and NF-*κ*B and stimulates HIV-1 transcription in promonocytic cells and primary macrophages. *The Journal of Biological Chemistry*.

[90] Roux P, Alfieri C, Hrimech M, Cohen EA, Tanner JE (2000). Activation of transcription factors NF-*κ*B and NF-IL-6 by human immunodeficiency virus type 1 protein R (Vpr) induces interleukin-8 expression. *Journal of Virology*.

[91] Sawaya BE, Khalili K, Gordon J, Taube R, Amini S (2000). Cooperative interaction between HIV-1 regulatory proteins Tat and Vpr modulates transcription of the viral genome. *The Journal of Biological Chemistry*.

[92] Kumar A, Manna SK, Dhawan S, Aggarwal BB (1998). HIV-Tat protein activates c-Jun N-terminal kinase and activator protein-1. *Journal of Immunology*.

[93] Muthumani K, Choo AY, Zong W (2006). The HIV-1 Vpr and glucocorticoid receptor complex is a gain-of-function interaction that prevents the nuclear localization of PARP-1. *Nature Cell Biology*.

[94] Lenassi M, Cagney G, Liao M (2010). HIV Nef is secreted in exosomes and triggers apoptosis in bystander CD4^+^ T cells. *Traffic*.

[95] Das SR, Jameel S (2005). Biology of the HIV Nef protein. *Indian Journal of Medical Research*.

[96] Olivetta E, Percario Z, Fiorucci G (2003). HIV-1 Nef induces the release of inflammatory factors from human monocyte/macrophages: involvement of Nef endocytotic signals and NF-*κ*B activation. *Journal of Immunology*.

[97] Mangino G, Percario ZA, Fiorucci G (2007). In vitro treatment of human monocytes/macrophages with myristoylated recombinant Nef of human immunodeficiency virus type 1 leads to the activation of mitogen-activated protein kinases, I*κ*B kinases, and interferon regulatory factor 3 and to the release of beta interferon. *Journal of Virology*.

[98] Varin A, Manna SK, Quivy V (2003). Exogenous Nef protein activates NF-*κ*B, AP-1, and c-Jun N-terminal kinase and stimulates HIV transcription in promonocytic cells: role in AIDS pathogenesis. *The Journal of Biological Chemistry*.

[99] Decrion AZ, Dichamp I, Varin A, Herbein G (2005). HIV and inflammation. *Current HIV Research*.

[100] Croft M, Benedict CA, Ware CF (2013). Clinical targeting of the TNF and TNFR superfamilies. *Nature Reviews Drug Discovery*.

[101] Knight DM, Trinh H, Le J (1993). Construction and initial characterization of a mouse-human chimeric anti-TNF antibody. *Molecular Immunology*.

[102] Goel N, Stephens S (2010). Certolizumab pegol. *MAbs*.

[103] Aukrust P, Liabakk N, Müller F, Lien E, Espevik T, Frøland SS (1994). Serum levels of tumor necrosis factor-*α* (TNF*α*) and soluble TNF receptors in human immunodeficiency virus type 1 infection—correlations to clinical, immunologic, and virologic parameters. *Journal of Infectious Diseases*.

[104] Whalen C, Horsburgh CR, Hom D, Lahart C, Simberkoff M, Ellner J (1995). Accelerated course of human immunodeficiency virus infection after tuberculosis. *American Journal of Respiratory and Critical Care Medicine*.

[105] Dezube BJ, Lederman MM, Chapman B (1997). The effect of tenidap on cytokines, acute-phase proteins, and virus load in human immunodeficiency virus (HIV)-infected patients: correlation between plasma HIV-1 RNA and proinflammatory cytokine levels. *Journal of Infectious Diseases*.

[106] Valdez H, Lederman MM (1997). Cytokines and cytokine therapies in HIV infection. *AIDS Clinical Review*.

[107] Marriott JB, Cookson S, Carlin E (1997). A double-blind placebo-controlled phase II trial of thalidomide in asymptomatic HIV-positive patients: clinical tolerance and effect on activation markers and cytokines. *AIDS Research and Human Retroviruses*.

[108] Wallis RS, Nsubuga P, Whalen C (1996). Pentoxifylline therapy in human immunodeficiency virus-seropositive persons with tuberculosis: a randomized, controlled trial. *Journal of Infectious Diseases*.

[109] Haraguchi S, Day NK, Kamchaisatian W (2006). LMP-420, a small-molecule inhibitor of TNF-alpha, reduces replication of HIV-1 and Mycobacterium tuberculosis in human cells. *AIDS Research and Therapy*.

[110] Ting PT, Koo JY (2006). Use of etanercept in human immunodeficiency virus (HIV) and acquired immunodeficiency syndrome (AIDS) patients. *International Journal of Dermatology*.

[111] Calabrese LH, Zein N, Vassilopoulos D (2004). Safety of antitumour necrosis factor (anti-TNF) therapy in patients with chronic viral infections: hepatitis C, hepatitis B, and HIV infection. *Annals of the Rheumatic Diseases*.

[112] Sha BE, Ogata-Arakaki DM, Fox L (2002). Effect of etanercept (enbrel) on interleukin 6, tumor necrosis factor, and markers of immune activation in HIV-infected subjects receiving interleukin 2. *AIDS Research and Human Retroviruses*.

[113] Wallis RS, Kyambadde P, Johnson JL (2004). A study of the safety, immunology, virology, and microbiology of adjunctive etanercept in HIV-1-associated tuberculosis. *AIDS*.

[114] Walker RE, Spooner KM, Kelly G (1996). Inhibition of immunoreactive tumor necrosis factor-*α* by a chimeric antibody in patients infected with human immunodeficiency virus type 1. *Journal of Infectious Diseases*.

[115] Gaylis N (2003). Infliximab in the treatment of an HIV positive patient with Reiter’s syndrome. *Journal of Rheumatology*.

[116] Cepeda EJ, Williams FM, Ishimori ML, Weisman MH, Reveille JD (2008). The use of anti-tumour necrosis factor therapy in HIV-positive individuals with rheumatic disease. *Annals of the Rheumatic Diseases*.

[117] Wang C, Wen T, Routy J, Bernard NF, Sekaly RP, Watts TH (2007). 4-1BBL induces TNF receptor-associated factor 1-dependent Bim modulation in human T cells and is a critical component in the costimulation-dependent rescue of functionally impaired HIV-specific CD8 T cells. *Journal of Immunology*.

[118] Yu Q, Yue FY, Gu XX, Schwartz H, Kovacs CM, Ostrowski MA (2006). OX40 ligation of CD4^+^ T cells enhances virus-specific CD8^+^ T cell memory responses independently of IL-2 and CD4^+^ T regulatory cell inhibition. *Journal of Immunology*.

[119] Cannons JL, Lau P, Ghumman B (2001). 4-1BB ligand induces cell division, sustains survival, and enhances effector function of CD4 and CD8 T cells with similar efficacy. *Journal of Immunology*.

[120] Taraban VY, Rowley TF, O'Brien L (2002). Expression and costimulatory effects of the TNF receptor superfamily members CD134 (OX40) and CD137 (4-1BB), and their role in the generation of anti-tumor immune responses. *European Journal of Immunology*.

[121] Watts TH (2005). TNF/TNFR family members in costimulation of T cell responses. *Annual Review of Immunology*.

[122] Serghides L, Bukczynski J, Wen T (2005). Evaluation of OX40 ligand as a costimulator of human antiviral memory CD8 T cell responses: comparison with B7.1 and 4-1BBL. *Journal of Immunology*.

[123] Ascierto PA, Simeone E, Sznol M, Fu Y, Melero I (2010). Clinical experiences with anti-CD137 and anti-PD1 therapeutic antibodies. *Seminars in Oncology*.

[124] Lee S, Salek-Ardakani S, Mittler RS, Croft M (2009). Hypercostimulation through 4-1BB distorts homeostasis of immune cells. *Journal of Immunology*.

[125] Harrison JM, Bertram EM, Boyle DB, Coupar BEH, Ranasinghe C, Ramshaw IA (2006). 4-1BBL coexpression enhances HIV-specific CD8 T cell memory in a poxvirus prime-boost vaccine. *Vaccine*.

[126] Chun T, Fauci AS (1999). Latent reservoirs of HIV: obstacles to the eradication of virus. *Proceedings of the National Academy of Sciences of the United States of America*.

[127] Chomont N, El-Far M, Ancuta P (2009). HIV reservoir size and persistence are driven by T cell survival and homeostatic proliferation. *Nature Medicine*.

[128] Abbas W, Herbein G (2012). Molecular understanding of HIV-1 latency. *Advances in Virology*.

[129] Withers DR, Jaensson E, Gaspal F (2009). The survival of memory CD4^+^ T cells within the gut lamina propria requires OX40 and CD30 signals. *Journal of Immunology*.

[130] Chun T, Engel D, Mizell SB, Ehler LA, Fauci AS (1998). Induction of HIV-1 replication in latently infected CD4^+^ T cells using a combination of cytokines. *Journal of Experimental Medicine*.

[131] Reuse S, Calao M, Kabeya K (2009). Synergistic activation of HIV-1 expression by deacetylase inhibitors and prostratin: implications for treatment of latent infection. *PLoS ONE*.

[132] Chan JKL, Greene WC (2011). NF-*κ*B/Rel: agonist and antagonist roles in HIV-1 latency. *Current Opinion in HIV and AIDS*.

[133] Burnett JC, Lim K, Calafi A, Rossi JJ, Schaffer DV, Arkin AP (2010). Combinatorial latency reactivation for HIV-1 subtypes and variants. *Journal of Virology*.

[134] Quivy V, Adam E, Collette Y (2002). Synergistic activation of human immunodeficiency virus type 1 promoter activity by NF-*κ*B and inhibitors of deacetylases: potential perspectives for the development of therapeutic strategies. *Journal of Virology*.

[135] Vandergeeten C, Quivy V, Moutschen M, van Lint C, Piette J, Legrand-Poels S (2007). HIV-1 protease inhibitors do not interfere with provirus transcription and host cell apoptosis induced by combined treatment TNF-*α* + TSA. *Biochemical Pharmacology*.

[136] Demonté D, Quivy V, Colette Y, van Lint C (2004). Administration of HDAC inhibitors to reactivate HIV-1 expression in latent cellular reservoirs: implications for the development of therapeutic strategies. *Biochemical Pharmacology*.

[137] El Kharroubi A, Piras G, Zensen R, Martin MA (1998). Transcriptional activation of the integrated chromatin-associated human immunodeficiency virus type 1 promoter. *Molecular and Cellular Biology*.

[138] Berger SL (2002). Histone modifications in transcriptional regulation. *Current Opinion in Genetics and Development*.

[139] Adam E, Quivy V, Bex F (2003). Potentiation of tumor necrosis factor-induced NF-kappa B activation by deacetylase inhibitors is associated with a delayed cytoplasmic reappearance of I kappa B alpha. *Molecular and Cellular Biology*.

[140] Bouchat S, Gatot J, Kabeya K (2012). Histone methyltransferase inhibitors induce HIV-1 recovery in resting CD4^+^ T cells from HIV-1+ HAART-treated patients. *AIDS*.

[141] Wolschendorf F, Bosque A, Shishido T (2012). Kinase control prevents HIV-1 reactivation in spite of high levels of induced NF-kappaB activity. *Journal of Virology*.

[142] Cannons JL, Hoeflich KP, Woodgett JR, Watts TH (1999). Role of the stress kinase pathway in signaling via the T cell costimulatory receptor 4-1BB. *Journal of Immunology*.

[143] Deeks SG, Autran B, Berkhout B (2012). Towards an HIV cure: a global scientific strategy. *Nature Reviews Immunology*.

